# *Haloferax profundi* sp. nov. and *Haloferax marisrubri* sp. nov., Isolated from the Discovery Deep Brine-Seawater Interface in the Red Sea

**DOI:** 10.3390/microorganisms8101475

**Published:** 2020-09-25

**Authors:** Guishan Zhang, Xiaoyan Dong, Yingjiao Sun, André Antunes, Tyas Hikmawan, Mohamed Fauzi Haroon, Junru Wang, Ulrich Stingl

**Affiliations:** 1 Red Sea Research Center, King Abdullah University of Science and Technology, Thuwal Jeddah 23955-6900, Saudi Arabia; zhangguishan@caas.cn (G.Z.); aglantunes@must.edu.mo (A.A.); tyas.hikmawan@kaust.edu.sa (T.H.); fauziharoon@gmail.com (M.F.H.); 2 Key Laboratory of Microbial Resources Collection and Preservation, Ministry of Agriculture, Institute of Agricultural Resources and Regional Planning, Chinese Academy of Agricultural Sciences, Beijing 100081, China; S1153501575@163.com (Y.S.); 202031200022@mail.bnu.edu.cn (J.W.); 3 Jiangsu Provincial Key Lab for Organic Solid Waste Utilization, National Engineering Research Center for Organic-based Fertilizers, Jiangsu Collaborative Innovation Center for Solid Organic Waste Resource Utilization, Nanjing Agricultural University, Nanjing 210095, China; 2016203022@njau.edu.cn; 4 State Key Laboratory of Lunar and Planetary Sciences, Macau University of Science and Technology, Taipa, Macau SAR, China; 5 Department of Microbiology and Cell Sciences, Fort Lauderdale Research and Education Center, UF/IFAS, University of Florida, Davie, FL 33314, USA

**Keywords:** *Haloferax profundi*, *Haloferax marisrubri*, halophilic archaeon, brine pool

## Abstract

Two extremely halophilic archaeal strains, designated SB29^T^ and SB3^T^, were isolated from the brine-seawater interface of Discovery Deep in the Red Sea. Cells of both strains were pleomorphic (irregular polyhedrals, ovals, and rods) and stained Gram-negative; colonies were pigmented pink. The sequence similarity of the 16S rRNA gene of strain SB29^T^ with that of its most closely related validly described species (*Hfx. sulfurifontis* DSM 16227^T^) and that of strain SB3^T^ with its closest validly described relative (*Hfx. denitrificans* ATCC 35960^T^) was 98.1% and 98.6%, respectively. The incomplete draft genomes of SB29^T^ and SB3^T^ are 3,871,125 bp and 3,904,985 bp in size, respectively, and their DNA G + C contents are 60.75% and 65.64%, respectively. The highest ANI values between the genomes of SB29^T^ and SB3^T^ and the most closely related genomes in GenBank were determined as 82.6% (*Hfx. sulfurifontis* ATCC BAA-897^T^, GenBank accession no. GCA_000337835.1) and 92.6% (*Haloferax denitrificans* ATCC 35960^T^, GenBank accession no. GCA_000337795.1), respectively. These data indicate that the two new isolates cannot be classified into any recognized species of the genus *Haloferax*, and, therefore, two novel species of the genus *Haloferax* are proposed: *Haloferax profundi* sp. nov. (type strain SB29^T^ = JCM 19567^T^ = CGMCC 1.14960^T^) and *Haloferax marisrubri* sp. nov. (type strain SB3^T^ = JCM 19566^T^ = CGMCC 1.14958^T^).

## 1. Introduction

Many studies indicated that deep-sea brine pools harbor distinctive microbial communities [1,2,3]. The isolation and characterization of novel extremophiles from brine environments will significantly advance our understanding of the in situ metabolisms, energy-producing processes, and environmentally adaptive activities in these harsh environments. The new strains in this study were isolated from the brine-seawater interface of Discovery Deep in the Red Sea, at a depth of 2000–2060 m, and formed two separate branches within the genus *Haloferax.*

Currently, there are 13 recognized species of *Haloferax* in the family *Haloferacaceae* of the order *Haloferacales,* according to the reclassification of the class Halobacteria into three orders and three families by Gupta [4]: *Hfx. volcanii* [5], *Hfx. mediterranei* [6], *Hfx. denitrificans* [7], *Hfx. gibbonsii* [8], *Hfx. alexandrinus* [9], *Hfx. lucentense* [10], *Hfx. sulfurifontis* [11], *Hfx. prahovense* [12], *Hfx. larsenii* [13], *Hfx. elongans*, *Hfx. mucosum* [14], *Hfx. chudinovii* [15], and *Hfx. namakaokahaiae* [16]. These 13 species were isolated from solar salterns, salt lakes, Permian potassium salt deposits, microbial mats from Hamelin Pool (Shark Bay, Australia), and sulfide- and sulfur-rich springs. No species of *Haloferax* had been previously isolated from deep-sea brine pool environments. Here, we describe the physiological and genomic properties of two novel strains within the genus *Haloferax* isolated from a brine-seawater interface, and propose to delineate them as two novel species. 

## 2. Materials and Methods

### 2.1. Isolation and Growth of Strains SB29^T^ and SB3^T^

Strains SB29^T^ and SB3^T^ were isolated using procedures described by Zhang et al. [17], and the two strains were grown aerobically in a modified R2A (MR2A) medium [18] for all physiological-chemical tests. The media contained the following ingredients (L^−1^): 0.5 g casamino acids (Difco), 0.5 g yeast extract (Difco), 0.5 g sodium pyruvate, 0.5 g peptone, 0.5 g glucose, 3.0 g trisodium citrate, 2.0 g KCl, 0.3 g K_2_HPO_4_, 0.5 g CaCl_2_, 20.0 g MgSO_4_∙7H_2_O, 200.0 g NaCl, and the pH was adjusted to 7.0–7.2.

### 2.2. Selection, Morphology and Phylogeny of Strains

In our cultivation experiment, we isolated 15 strains that formed two groups based on 16S rRNA gene sequences and colony and cell morphologies. Eight strains from the Discovery interface 1 (Di1), designated strains SB1 to SB8, formed small (1 mm), pink, translucent, convex colonies on an agar medium. This group of strains all consisted of pleomorphic rods (Supplementary Figures S1 and S2), and an initial analysis of their partial 16S rRNA gene sequences indicated they were most closely related to *Hfx. denitrificans* ATCC 35960^T^ (98.6% similarity). Strain SB3^T^ was selected as a representative strain for further taxonomic characterization. Seven strains isolated from the Discovery interface 2 (Di2), designated SB22–25 and SB28–30, formed pinkish-red, opaque, domed, mucoid colonies, 3–5 mm in diameter, containing pleomorphic cells. Analyses of their partial 16S rRNA gene sequences indicated they were most closely related to *Hfx. sulfurifontis* (98.1% similarity). Strain SB29^T^ was selected as a representative strain for further taxonomic characterization.

### 2.3. Phenotypic Tests

**Figure 1:**
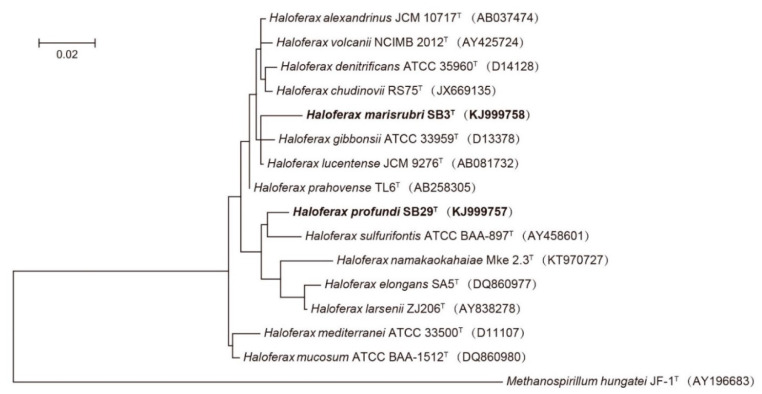
Maximum likelihood (ML) phylogenetic tree based on 16S rRNA gene sequences showing the relationships between strain SB3^T^ and SB29^T^ and closely related taxa. Scale bar indicates 0.01 substitutions per nucleotide position. *Methanospirillum hungatei* JF-1^T^ (AY196683) was used as outgroup. GenBank accession numbers are listed for each sequence in parentheses.

Phenotypic tests of SB29^T^ and SB3^T^ were performed according to the proposed minimum standards for the description of novel taxa in the order *Halobacteriales* [19]. Of the 13 published type strains, 12 were selected as reference strains for physiological tests (Table 1). *Hfx. namakaokahaiae* was described after our experiments were already completed. As *Hfx. namakaokahaiae* did not represent one of the closest relatives of either novel strain (Figure 1), we amended Table 1 with data from McDuff et al. [16]. Cell morphology and motility in exponentially growing cultures in the liquid MR2A medium were examined using a Leica microscope equipped with phase-contrast optics (Leica DM 6000 B). The minimal salt concentration required to prevent cell lysis was tested by suspending washed cells in serial sterile saline solutions containing NaCl ranging from 0 to 15.0% (*w*/*v*), and the stability of the cells was observed by light microscopy. Sample preparation for transmission electron microscopy (TEM) was based on previously published methods [20,21] and the cells were analyzed on a Technai 12 transmission electron microscope (FEI Company, Hillsboro, OR, USA). Gram-staining was performed using the method outlined by Dussault [22]. Most biochemical and nutritional tests were performed as described by Allen et al. [14], Xu et al. [13], and Goh et al. [23]. In brief, anaerobic archaeal growth and gas formation with nitrate as an electron acceptor were measured in 10 mL stoppered tubes filled with 4 mL media containing NaNO_3_ (5.0 g L^−1^), L-arginine (5.0 g L^−1^), and DMSO (5.0 g L^−1^). An inverted Durham tube was added to capture the gas production; nitrite formation was monitored colorimetrically. The optimal growth temperature was determined after incubation on MR2A agar and shaking in MR2A liquid medium at 10, 15, 20, 25, 28, 30, 33, 37, 40, 45, and 50 °C. Growth was measured as an increase in turbidity at 660 nm, determined in a spectrophotometer. Tolerance to NaCl concentration was tested on both MR2A agar and liquid media with 0.5, 1.0, 1.2, 1.5, 2.0, 3.0, 4.0, 4.3, 4.5, 5.0, 5.5, 5.8, and 6.0 M NaCl added. Similarly, tolerance to MgCl_2_ was tested on both MR2A agar liquid media containing 0, 0.05, 0.1, 0.2, 0.3, 0.4, 0.5, 0.7, 0.75, and 1.0 M MgCl_2_. pH tolerance of the strains SB29^T^ and SB3^T^ was tested in an MR2A liquid medium, adjusted to pH 5.5 to 10.0 with 20 mM of the following buffer systems: MES (pH 5.5 and 6.0), PIPES (pH 6.5 and 7.0), HEPES (pH 7.5 and 8.0), Tricine (pH 8.5), and CAPSO (pH 9.0, 9.5 and 10.0). Oxidase activity was tested using the oxidase reagent kit (bioMérieux) according to the manufacturer’s instructions. Catalase activity was determined by pouring 3.0% H_2_O_2_ solution onto bacterial colonies and observing bubble production. Esterase activity was detected as outlined by Gutíérrez and González [24].

Isolates and reference strains were grown in tubes containing an MR2A liquid medium supplemented with 0.5% (*w*/*v*) Na_2_S_2_O_3_, and H_2_S production was analyzed using a C2V-200 micro gas chromatograph from Thermo Scientific. Acid production was determined using the API 50 CHB system (bioMérieux), where the API 50 CHB medium was supplemented with (L^−1^): 2.0 g KCl, 0.3 g K_2_HPO_4_, 0.5 g CaCl_2_, 20.0 g MgSO_4_∙7H_2_O, and 180.0 g NaCl. The pH was maintained between 7.0–7.2. The neutral oligotrophic haloarchaeal medium contained the following ingredients (L^−1^): 0.05 g yeast extract, 5.4 g KCl, 0.3 g K_2_HPO_4_, 0.25 g CaCl_2_, 0.25 g NH_4_Cl, 26.8 g MgSO_4_ ∙ 7H_2_O, 23.0 g MgCl_2_∙ 6H_2_O, and 180.0 g NaCl (pH adjusted to 7.0–7.2 with a 1.0 M NaOH solution). This medium was combined with single carbon sources (5.0 g L^−1^) to test the hydrolysis of casein, starch, gelatin, and Tween 80 [25,26]. Biochemical activities and the use of organic substrates as sole carbon and energy sources were also evaluated using API 20E and API 20NE kits (bioMérieux) and Biolog GEN III MicroPlates (bioMérieux), according to the manufacturers’ instructions, with the exception of a salinity adjustment to 20% (*w*/*v*) NaCl and supplementing the media with (L^−1^) 2.0 g KCl, 0.3 g K_2_HPO_4_, 0.5 g CaCl_2,_ and 20.0 g MgSO_4_∙7H_2_O (pH between 7.0–7.2). Susceptibility to antibiotics was assessed on MR2A agar using the Kirby–Bauer disc diffusion method according to Gutíérrez et al. [11] and Du et al. [27]. Generation times for SB29^T^ and SB3^T^ were determined at 33 °C and 37 °C, respectively, by the method of Robinson et al. [28]. The NaCl, pH, and temperature ranges for growth, sensitivity to antibiotics, and use of carbon sources are presented in the species descriptions. The characteristics that distinguish strains SB29^T^ and SB3^T^ from other validly described *Haloferax* species are shown in Table 1. Whole-cell protein profiles determined by sodium dodecyl sulfate polyacrylamide gel electrophoresis (SDS-PAGE) [29] were used as a rapid method for distinguishing between bacterial species [30]. Cells of strain SB29^T^ and SB3^T^ were grown in an MR2A liquid medium at 37 °C and were harvested at the late exponential growth phase for characterization of polar lipids. Polar lipids were extracted from 200 mg of freeze-dried cell material using a chloroform/methanol (0.3%, *w*/*v*)/aqueous NaCl mixture (1:2:0.8, *v*/*v*/*v*), modified after Bligh and Dyer [31], and were recovered into the chloroform phase by adjusting the mixture to a ratio of 1:1:0.9 (*v*/*v*/*v*) and subsequently separated by two-dimensional silica gel thin-layer chromatography (silica gel GF254, 0.25-mm thick, Haiyang Chemical Co., Qingdao, China). The first direction was developed in a chloroform/methanol/water (65:25:4, *v*/*v*/*v*) mixture and the second was developed in a chloroform/methanol/acetic acid/water (80:12:15:4, *v*/*v*/*v*/*v*) mixture. Total lipid material was detected using staining reagents (phosphomolybdic acid, molybdenum blue, ninhydrin, and α-naphthol) specific for defined functional groups [32]. Polar lipid analysis was performed by the identification services at the China Center of Industrial Culture Collection in Beijing, China. The analysis of respiratory quinones in strain SB29^T^ and its closest relative, *Hfx. sulfurifontis* DSM 16227^T^, as well as strain SB3^T^ and its closest relative, *Hfx. denitrificans* ATCC 35960^T^, was performed by the identification service of the DSMZ, Braunschweig, Germany.

### 2.4. Genomic Analyses

Genomic DNA was extracted using a commercial kit (TaKaRa MiniBEST Bacteria Genomic DNA Extraction Kit V 3.0) and subsequently sequenced on the Illumina HiSeq 2000 platform at the Bioscience Core Lab at the King Abdullah University of Science and Technology (KAUST, Saudi Arabia). Filtering and trimming of the raw data was performed using PRINSEQ v0.20.4 [33]. SOAPdenovo v1.05 [34,35] with default parameters was used to assemble the trimmed reads. The size of the genome was estimated by k-mer analysis [36] and genome completeness was determined with CheckM v1.0.3 [37]. Calculation of the DNA G + C content and prediction of protein-coding open reading frames (ORFs) was done in Glimmer v3.02 [38]. We used RNAmmer v1.2 to predict rRNAs [39] and tRNAscan-SE v1.21 to predict tRNAs [40]. Genome completeness was determined by CheckM v1.0.3.

The DNA–DNA relatedness value is a standard method for defining individual species, with 70% being the recommended minimum relatedness value for the DNA of strains of the same species [41,42]. Digital DNA–DNA hybridization (DDH) values were obtained by means of genome-to-genome sequence comparison via GGDC 2.0 using Formula (2) as implemented in the software [43]. Furthermore, as a complement to DNA–DNA hybridization [44], average nucleotide identity (ANI) values of the total genomic sequences shared between the genomic sequences of strain SB29^T^ or SB3^T^ with closely related genomic sequences from GenBank were determined according to Goris et al. [45]. Whole-genome sequences in a pairwise comparison were split into consecutive 1000 bp windows. Sequences were aligned with nucmer in MUMmer v3.23 [46] and ANI values were calculated using JSpeciesWS [47].

### 2.5. Phylogenetic Analyses

Multiple sequence alignments of 16S rRNA gene sequences of strains SB29^T^ and SB3^T^ with those of their most closely related taxa were performed using CLUSTALX v1.81 [48]. A phylogenetic tree was constructed with the maximum likelihood (ML) method using MEGA v6.0 [49], based on recommendations by Minegishi et al. (2012) [50], and sequences of strains SB29^T^ and SB3^T^ were placed within the genus *Haloferax* (Figure 1). Maximum parsimony (MP) and neighbor-joining (NJ) methods were additionally used to analyze and verify the taxonomic positions of the novel isolates and reference strains (Supplementary Figures S5 and S6). All available genome sequences of validly described *Haloferax* species (11 genomes) were retrieved from NCBI and re-annotated using PROKKA [51]. We used Roary to identify the core genes of these type strains [52] and a multiple sequence alignment of 31 single-copy core genes was obtained using MAFFT [53]. A phylogenetic tree based on the concatenation of these single-copy core genes was inferred using the neighbor-joining (NJ) method and rooted with sequences retrieved from *Methanospirillum hungatei* JF-1^T^ (Supplementary Figure S7).

### 2.6. Data Availability

The GenBank/EMBL/DDBJ accession numbers for the 16S rRNA genes of strains SB29T and SB3T are KJ999757 and KJ999758, respectively. The whole genome shotgun projects were deposited at DDBJ/EMBL/GenBank under the accession identifications LOPV00000000 and LOPW00000000 for strains SB29T and SB3T, respectively. Phase-contrast micrographs of the late exponential phase of strains SB29T and SB3T cells, sodium dodecyl sulfate polyacrylamide gel electrophoresis patterns of the whole-cell proteins, thin-layer chromatograms of the polar lipids extracted from strains SB29T and SB3T, additional phylogenetic trees, and a table of average nucleotide identity values with closely related strains are available as Supplementary Materials.

## 3. Results

All phylogenetic analyses are in agreement and show that both strains branch separately from the existing validly described species of the genus *Haloferax*, indicating that these strains represent novel species (Figure 1, Figures S6 and S7). Based on the results of the genomic assembly, both genomes are estimated to be 99.0% complete. The draft genome of SB29^T^ is 3,871,125 bp in size with 771 contigs, 57 scaffolds, and a 136-fold coverage; the draft genome of strain SB3^T^ is 3904,985 bp in size with 66 contigs, 26 scaffolds, and a 154-fold coverage. A total of 4053 ORFs, 47 tRNAs, and 2 rRNAs are predicted for strain SB29^T^, and a total of 3824 ORFs, 51 tRNAs, and 2 rRNAs are predicted for strain SB3^T^. The DNA G + C contents for strain SB29^T^ and SB3^T^ are 60.75% and 65.64%, respectively, which are very similar to those reported for other *Haloferax* species (Table 1). The genomic data suggests that both strains SB29^T^ (1473 bp) and SB3^T^ (1446 bp) only possess one copy of the 16S rRNA gene.

Strains SB29^T^ and SB3^T^ had low DNA–DNA relatedness and DDH with each other and with all recognized *Haloferax* species (Table 1, Tables S1 and S2). An ANI boundary of 95–96% is recommended to taxonomically circumscribe prokaryotic species [54,55]. The highest ANI values between the genomes of SB29^T^ and SB3^T^ and their closely related genomes in GenBank were determined as 82.6% (*Hfx. sulfurifontis* ATCC BAA-897^T^, GenBank accession no. GCA_000337835.1) and 92.6% (*Haloferax denitrificans* ATCC 35960^T^, GenBank accession no. GCA_000337795.1), respectively (Supplementary Tables S1 and S2). This further indicates that strains SB29^T^ and SB3^T^ represent two different novel species.

Phylogenetic, phenotypic, and chemotaxonomic data indicate that strains SB29^T^ and SB3^T^ are members of the genus *Haloferax*. The DNA–DNA hybridization data, whole-cell protein profiles, and phenotypic characteristics (Table 1) justify the creation of two novel species within the genus *Haloferax* to accommodate these strains, for which the names *Haloferax profundi* sp. nov. and *Haloferax marisrubri* sp. nov., respectively, are proposed. The major respiratory quinones of strain SB29^T^ were MK8 (69%) and MK8 (VIII-H2, 31%), while those of SB3^T^ were MK8 (90%) and MK8 (VIII-H2, 10%), which differ from other closely related species of the same genus *Haloferax*, i.e., *Hfx. sulfurifontis* DSM 16227^T^ (MK8, 74%, and MK8 (VIII-H2, 26%)) and *Hfx. denitrificans* ATCC 35960^T^ (MK8, 81%, and MK8 (VIII-H2, 19%)).

Whole-cell protein analysis of strains SB29T and SB3T with various other *Haloferax* strains using SDS-PAGE (Supplementary Figure S3) showed complex band patterns, but clear differences between the novel strains reported here and validly described *Haloferax* species.

Both strains contained phosphatidyl glycerol (PG), phosphatidyl glycerorophosphate-methyl ester (PGP-Me), sulfated mannosyl glucosyl diether (S-DGD-1), and mannosyl glucosyldiether (DGD-1), but lacked phosphatidyl glycerosulfate (PGS), which is a characteristic of other species of the genus *Haloferax* [56] (Supplementary Figure S4).

## 4. Discussion

Based on the phenotypic, phylogenetic, and genetic differences between strains SB29^T^ and SB3^T^ and their most closely related species, we propose to delineate them as two novel species, *Haloferax profundi* sp. nov. and *Haloferax marisrubri* sp. nov.

### 4.1. Description of Haloferax profundi sp. nov.

*Haloferax profundi* (pro.fun’di. L. gen. n. profundi, from the depths of the sea). Cells are Gram-negative, motile, and pleomorphic, shaped as irregular polyhedra, ovals, and rods (average size of 0.3–0.6 × 1.0–6.8 μm); under optimal conditions, cells undergo exponential growth and elongate up to 13 μm (Supplementary Figures S1 and S2). Colonies on a complex agar medium formed pinkish-red, opaque, domed colonies, with a diameter of 3–5 mm. Growth occurs in 1.0–5.5 M NaCl (optimum between 3.5–4.5 M NaCl), at 15–45 °C (optimum at 33 °C), and at pH 5.5–9.0 (optimum between pH 7.0–7.5). Growth occurs in media containing between 0.05–0.7 M Mg^2+^ (optimum at 0.2 M Mg^2+^), and cell lysis occurs in solutions below 0.9 M NaCl. The generation time at 33 °C is 36 ± 4 min. Cells are chemoorganotrophic, aerobic, oxidase, and catalase positive, and esterase negative. Nitrate is not reduced to nitrite, nor is any gas produced from nitrate. Indole is produced from tryptophan, but H_2_S is not produced from thiosulfate. No anaerobic growth is observed with nitrate, DMSO, or L-arginine. Gelatin, casein, and starch are hydrolyzed, unlike Tween 80. The following substrates are used as carbon sources: D-glucose, D-mannitol, D-maltose, malate, citrate, glycerol, sucrose, and trehalose. Acid is produced from D-glucose, glycerol, D-maltose, sucrose, and trehalose. The following substrates are not used as carbon sources: D-sorbitol, d-arabitol, ethanol, fumarate, D-mannose, D-fructose, D-galactose, L-rhamnose, xylose, glycine, lactose, raffinose, L-histidine, L-lysine, or L-ornithine. Strain SB29^T^ contains phosphatidyl glycerol (PG), phosphatidyl glycerol phosphate-methylester (PGP-Me), sulfated mannosyl glucosyldiether (S-DGD-1), mannosyl glucosyldiether (DGD-1), two unidentified phospholipids (UPL1 and UPL2), and glycolipid (GL), but phosphatidyl glycerol sulfate (PGS) was absent. The major respiratory quinones of strain SB29^T^ are MK8 (69%) and MK8 (VIII-H2, 31%). The genome draft as presented here has a size of 3,871,125 bp and a DNA G + C content of 60.75 mol%. The type strain is SB29^T^ (=JCM 19567^T^ = CGMCC 1.14960^T^), its genome accession number is GCA_001469865.1, and it was isolated from the Discovery Deep brine-seawater (Di2) in the Red Sea.

### 4.2. Description of Haloferax marisrubri sp. nov.

*Haloferax marisrubri* (ma.ris.ru’bri. L. n. mare, maris, the sea; L. adj. ruber, red; N.L. gen. n. marisrubri, of the Red Sea). Cells are Gram-negative, motile, and pleomorphic, with irregular polyhedra, ovals, and rods (average, 0.4–0.7 × 1.0–5.3 μm). Under optimal conditions, growth is exponential and cells elongate to up to 11 μm (Supplementary Figures S1 and S2). Colonies on a complex agar medium are pink, translucent, convex, and about 1.0 mm in diameter. Growth occurs between 1.5–5.8 M NaCl (optimum between 4.5–5.0 M NaCl), at 20–50 °C (optimum at 37 °C), and between pH 6.5–9.0 (optimum between pH 7.5–8.0). Growth occurs in media containing 0.05–1.0 M Mg^2+^ (optimum at 0.35 M Mg^2+^), and cell lysis occurs in NaCl solutions below 0.95 M. The generation time at 37 °C is 47 ± 5 min. Cells are chemoorganotrophic, aerobic, oxidase, and catalase positive, and esterase negative. Nitrate is not reduced to nitrite, and no gas is produced from nitrate. Indole is not produced from tryptophan and H_2_S is not produced from thiosulfate. Anaerobic growth is not observed with nitrate, DMSO, or L-arginine. Gelatin and starch are hydrolyzed, unlike casein and Tween 80. The following substrates are used as carbon sources: D-glucose, D-mannitol, D-maltose, citrate, glycerol, and trehalose. Acid is produced from D-glucose, glycerol, D-maltose, and trehalose. The following substrates are not used as carbon sources: D-mannose, D-fructose, sucrose, D-sorbitol, D-arabitol, ethanol, malate, fumarate, D-galactose, L-rhamnose, xylose, glycine, lactose, raffinose, L-histidine, L-lysine, or L-ornithine. Strain SB3^T^ contains phosphatidyl glycerol (PG), phosphatidyl glycerol phosphate-methylester (PGP-Me), sulfated mannosyl glucosyldiether (S-DGD-1), mannosyl glucosyldiether (DGD-1), two unidentified phospholipids (UPL1 and UPL2), and glycolipid (GL), but phosphatidyl glycerol sulfate (PGS) is absent. The major respiratory quinones of SB3^T^ are MK8 (90.0%) and MK8 (VIII-H2, 10.0%). The genome draft, as presented here, has a size of 3,904,985 bp and a DNA G + C content of 65.64 mol%. The type strain is SB3^T^ (= JCM 19567^T^ = CGMCC 1.14960^T^), its genome accession number is GCA_001469875.2, and it was isolated from the Discovery Deep brine-seawater (Di1) in the Red Sea.

## 5. Conclusions

In this study, we isolated and described two novel strains of extremely halophilic Archaea from the brine-seawater interface of Discovery Deep in the Red Sea. Phylogenetic, phenotypic, and genetic analyses indicated that these strains represent two novel species of the genus *Haloferax*, for which we propose the names *Haloferax profundi* sp. nov. and *Haloferax marisrubri* sp. nov.

## Figures and Tables

**Table 1:** Phenotypic and other characteristics that distinguish the Discovery Deep brine-seawater interface isolates SB29^T^ and SB3^T^ from other Haloferax species. Taxa: 1, SB29^T^; 2, SB3^T^; 3, *Hfx. elongans* JCM 14791^T^ [14]; 4, *Hfx. mucosum* JCM 14792^T^ [14]; 5, *Hfx. mediterranei* JCM 8866^T^ [6]; 6, *Hfx. volcanii* JCM 8879^T^ [5]; 7, *Hfx. lucentense* DSM 14919^T^ [10]; 8, *Hfx. denitrificans* JCM 8864^T^ [7]; 9, *Hfx. gibbonsii* JCM 8863^T^ [8]; 10, *Hfx. alexandrinus* JCM 10717^T^ [9]; 11, *Hfx. sulfurifontis* DSM 16227^T^ [11]; 12, *Hfx. prahovense* DSM 18310^T^ [12]; 13, *Hfx. larsenii* JCM 13917^T^ [13]; 14, *Hfx. chudinovii* DSM 26526^T^ [15]; 15, *Hfx. namakaokahaiae* DSM 29988^T^ [16]. +, positive; −, negative; ±, variable; w, weakly positive; *Hfx.*, *Haloferax.*

Characteristic	1	2	3	4	5	6	7	8	9	10	11	12	13	14	15
Pigmentation	Pink	Pink-Red	Red	Pink-Red	Pink	Red-Orange	Pink	Orange-Red	Orange-Red	Red	Salmon-Pink	Beige-Orange	Orange-Red	Pink-Red	Red
Motility	+	+	Rotating	−	+	Rotating	+	−	+	−	−	−	+	−	−
NaCl range (M)	1.0–5.5	1.5–5.8	1.7–5.1	1.7–5.1	1.3–4.7	1.0–4.5	1.8–5.1	1.5–4.5	1.5–5.2	1.7–5.2	1.0–5.2	2.5–5.2	1.0–4.8	1.1–4.6	0.5–5.4
NaCl optimum (M)	3.5–4.5	4.5–5.0	2.6–3.4	2.6–3.4	2.9	1.7–2.5	4.3	2.0–3.0	2.5–4.3	4.3	2.1–2.6	3.5	2.2–3.4	2.5–3.0	1–2
Minimum Mg^2+^ (M)	0.2	0.35	0.2	0.2	0.02	0.02	0.07	0.06	0.2	0.33	0.001	0.1	0.005	0.02	nd
Temp. range (°C)	15–45	20–50	30–55	23–55	25–45	20–45	10–40	30–55	25–55	20–55	18–50	23–51	25–55	23–51	nd
Temp. optimum (°C)	33	37	53	42–53	35–37	45	37	50	35–40	37	32–37	38–48	42–45	40–45	30
pH range	5.5–9.0	6.5–9.0	7.0–9.0	6.0–10.0	5.5–8.0	6.0–8.0	5.0–9.0	6.0–8.0	5.0–8.0	5.5–7.5	5.0–9.0	6.0–8.5	6.0–8.5	5.5–8.0	nd ^#^
Oxidase test	+	+	±	−	+	+	+	+	+	+	+	+	+	±	+
H_2_S formation															
from thiosulfate	−	−	−	−	−	+	+	+	+	+	+	+	+	−	nd
Hydrolysis of:															
Gelatin	+	+	+	+	+	−	−	+	+	+	+	−	+	−	nd
Casein	+	−	+	+	+	−	−	−	+	−	−	−	−	−	nd
Starch	+	+	+	−	+	−	−	−	−	−	−	+	+	+	−
Tween 80	−	−	+	−	+	−	+	−	+	+	+	+	+	+	nd
Acid production from:															
D-glucose	−	−	−	−	−	+	−	−	+	+	−	+	−	−	nd
Mannose	−	−	−	−	+	−	−	−	+	−	−	+	−	−	nd
Galactose	−	−	−	−	+	+	−	+	+	−	+	−	−	−	nd
Xylose	−	−	−	−	+	+	+	−	+	+	+	−	−	+	nd
Sucrose	+	−	+	+	+	+	−	+	+	+	+	−	w	−	nd
DNA G + C content															
(mol%)	60.75	65.64	61.2	61.8	60.25	65.63	66.4	66.3	66.07	66.15	66.3	65.7	61.8	65.0 *	61.5 *
DNA-DNA reassociation (DDH):															
with SB29^T^ (%)	100	32.1	41.8	38.2	31.2	37.4	43.2	35.5	42.1	32.8	41.7	34.8	38.9	31.6	30.9
with SB3^T^ (%)	32.1	100	36.8	45.2	32.9	43.5	44.7	43.9	37.8	44.1	42.3	38.3	34.7	46.3	35.6

Notes: Data on temperature range (°C), temperature optimum (°C), and pH range for all strains, except for strain SB29^T^ and SB3^T^, were taken from references listed above. All other experiments listed in Table 1 were tested in parallel with *Hfx. elongans* JCM 14791^T^, *Hfx. mucosum* JCM 14792^T^, *Hfx. mediterranei* JCM 8866^T^, *Hfx. volcanii* JCM 8879^T^, *Hfx. lucentense* DSM 14919^T^, *Hfx. denitrificans* JCM 8864^T^, *Hfx. gibbonsii* JCM 8863^T^, *Hfx. alexandrinus* JCM 10717^T^, *Hfx. sulfurifontis* DSM 16227^T^, *Hfx. prahovense* DSM 18310^T^, *Hfx. larsenii* JCM 13917^T^, and *Hfx. chudinovii* DSM 26526^T^. Data for *Hfx. namakaokahaiae* DSM 29988^T^ were taken from McDuff et al. (2016) [16]. All DNA G + C content values (mol%) were calculated based on genomic data, except the ones marked with an asterisk (*) that were calculated using the Tm method. ^#^ pH optimum as reported: 6–8; nd: not determined.

## Supplementary Materials

The following are available online at https://www.mdpi.com/2076-2607/8/10/1475/s1, Figure S1. Phase-contrast micrograph of strain SB29^T^ (a) and SB3^T^ (b) after 72 h growth in liquid MR2A medium at 37 °C, illustrating the pleomorphic nature of cells of strains SB29^T^ and SB3^T^. Scale bars are shown on the images, Figure S2. Scanning Electron Microscope (SEM) micrograph of strain SB29^T^ (a) and SB3^T^ (b) after 72 h of growth in liquid MR2A medium at 37 °C, scale bar = 1 μm for panel a, scale bar = 2 μm for panel b, Figure S3. Whole-cell proteins from various halophilic archaeal strains and isolates SB29^T^ and SB3^T^, following separation by SDS-PAGE. Following cell lysis, approximately 15 µg of protein was applied to each lane. Proteins were stained with Coomassie brilliant blue. Lanes: 1, molecular mass marker; 2, Strain SB29^T^; 3, Strain SB3^T^; 4, *Hfx. alexandrinus* JCM 10717^T^; 5, *Hfx. larsenii* JCM 13917^T^; 6, *Hfx. lucentense* DSM14919^T^; 7, *Hfx. prahovense* DSM 18310^T^; 8, *Hfx. sulfurifontis* DSM 16227^T^; 9. *Hfx. elongans* JCM 14791^T^, Figure S4. Polar lipids profiles for strain SB3^T^ (a) and SB29^T^ (b) include PG, PGP-Me, S-DGD-1, DGD-1, UPL1 and UPL2; and an unidentified GL, which support the placement of both strains into the genus *Haloferax*. The TLC plates were stained with 10% phosphomolybdic acid in 95% ethanol for detection of total lipids, 0.2 % ninhydrin in 1-butanol for aminolipids, molybdenum blue reagent (Sigma) for phospholipids, and a-naphthol staining reagent (Sigma) for cholines, Figure S5. Neighbour-joining phylogenetic tree based on 16S rRNA gene sequences showing the relationships between strain SB3^T^ and SB29^T^ (bold) and related taxa. Scale bar indicates 0.01 substitutions per nucleotide position. *Methanospirillum hungatei* JF-1^T^ (AY196683) was used as outgroup. GenBank accession numbers are indicated for each strain in parentheses, Figure S6. Maximum parsimony phylogenetic tree based on 16S rRNA gene sequences showing the relationships between strain SB3^T^ and SB29^T^ (both in bold) and related taxa. *Methanospirillum hungatei* JF-1^T^ (AY196683) was used as outgroup. GenBank accession numbers are indicated for each strain in parentheses, Figure S7. Phylogenomic tree based on the concatenation of the 31 single-copy core genes from all 11 available genomes of type strains for *Haloferax* spp. were inferred with Neighbor-Joining (NJ) method and rooted by *Methanospirillum hungatei* JF-1^T^. The genes were katG2, top6A, ntpB, atpA, ftsZ_2, aroB, fusA, uvrA, Ion, gatA, ndk, rplB, ftsZ_1, pdxS, nikR_2, guaA_2, rpoB_1, rpsK, rpsJ_1, tufB_1, rps12P, rps28e, DUF2073, rps2P, rpoK, rpl40e, rps3p, Mcm2 and three hypothetical proteins (PRK00939, PRK04016, PRK04243; https://www.ncbi.nlm.nih.gov/cdd) as determined in Roary (see methods section), Table S1. Average nucleotide identity (ANIm) and digital DNA-DNA hybridization (dDDH) values (%) between the genomes of strain SB3^T^ (GenBank accession: LOPW00000000), and closely related genomes in GenBank, Table S2. Average nucleotide identity (ANIm) and digital DNA-DNA hybridization (dDDH) values (%) between the genomes of strain SB29^T^ (GenBank accession no.: LOPV00000000), and closely related genomes in GenBank.
