# Supplementary material for: Haloferax profundi sp. nov. and Haloferax marisrubri sp. nov., Isolated from the Discovery Deep Brine-Seawater Interface in the Red Sea
Source: Microorganisms. 2020 Sep 25;8(10):1475. doi: 10.3390/microorganisms8101475 (PMC7599478; doi:10.3390/microorganisms8101475)
Supplement: Supplementary file 1 [file microorganisms-08-01475-s001.pdf]

Supplementary Materials

Haloferax profundi sp. nov. and *Haloferax marisrubri* sp. nov., Isolated from the Discovery Deep Brine-Seawater Interface in the Red Sea

Guishan Zhang [1,2], Xiaoyan Dong [3], Yingjiao Sun [2], André Antunes [1,4], Tyas Hikmawan [1], Mohamed Fauzi Haroon [1], Junru Wang [2] and Ulrich Stingl [1,5,*]

[1] Red Sea Research Center, King Abdullah University of Science and Technology, Thuwal Jeddah 23955-6900, Saudi Arabia; zhangguishan@caas.cn (G.Z.); aglantunes@must.edu.mo (A.A.); tyas.hikmawan@kaust.edu.sa (T.H.); fauziharoon@gmail.com (M.F.H.)

[2] Key Laboratory of Microbial Resources Collection and Preservation, Ministry of Agriculture, Institute of Agricultural Resources and Regional Planning, Chinese Academy of Agricultural Sciences, Beijing 100081, China; S1153501575@163.com (Y.S.); 202031200022@mail.bnu.edu.cn (J.W.)

[3] Jiangsu Provincial Key Lab for Organic Solid Waste Utilization, National Engineering Research Center for Organic-based Fertilizers, Jiangsu Collaborative Innovation Center for Solid Organic Waste Resource Utilization, Nanjing Agricultural University, Nanjing 210095, China; 2016203022@njau.edu.cn

[4] State Key Laboratory of Lunar and Planetary Sciences, Macau University of Science and Technology, Taipa, Macau SAR, China

[5] Department of Microbiology and Cell Sciences, Fort Lauderdale Research and Education Center, UF/IFAS, University of Florida, Davie, FL 33314, USA

[*] Correspondence: ustingl@ufl.edu; Tel.: +1-954-577-6326

Supplementary Figures.

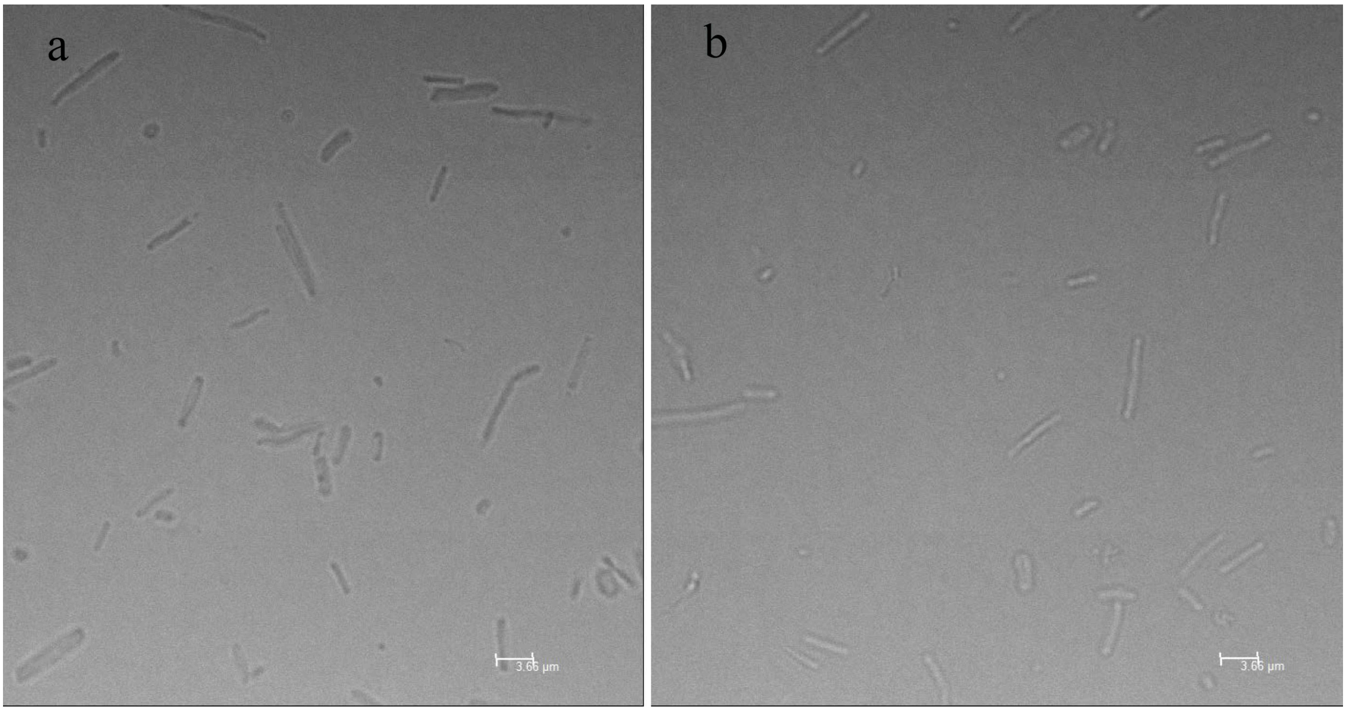

Figure S1. Phase-contrast micrograph of strain SB29^T (a) and SB3^T (b) after 72 h growth in liquid MR2A medium at 37 ℃, illustrating the pleomorphic nature of cells of strains SB29^T and SB3^T. Scale bars are shown on the images.

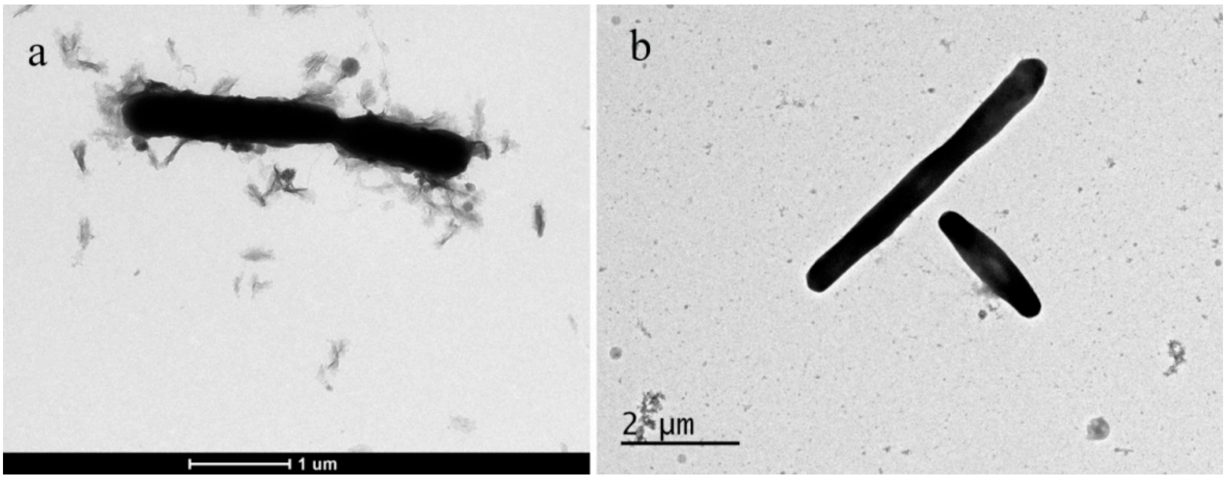

Figure S2. Scanning Electron Microscope (SEM) micrograph of strain SB29^T (a) and SB3^T (b) after 72 h of growth in liquid MR2A medium at 37 ºC, scale bar = 1 µm for panel a, scale bar = 2 µm for panel b.

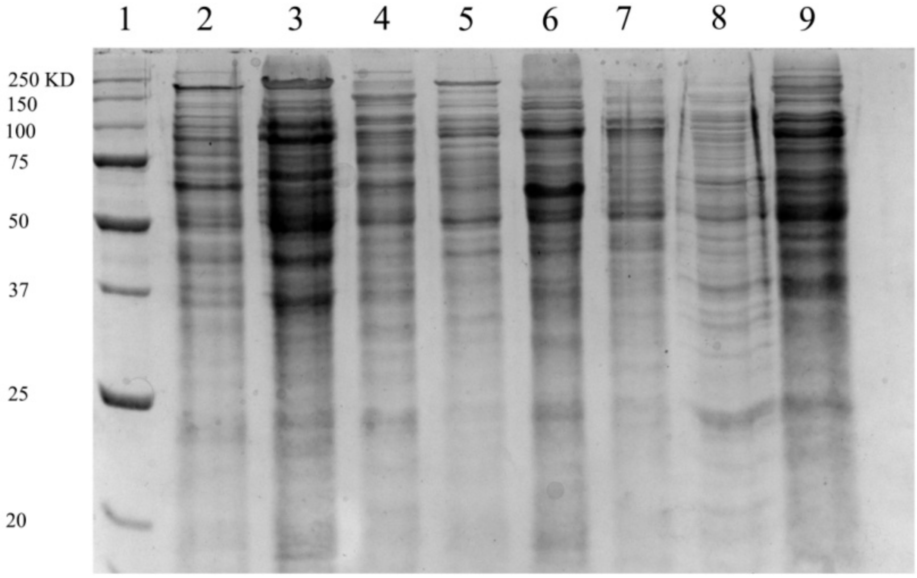

Figure S3. Whole-cell proteins from various halophilic archaeal strains and isolates SB29^T and SB3^T, following separation by SDS-PAGE. Following cell lysis, approximately 15 μg of protein was applied to each lane. Proteins were stained with Coomassie brilliant blue. Lanes: 1, molecular mass marker; 2, Strain SB29^T; 3, Strain SB3^T; 4, *Hfx. alexandrinus* JCM 10717^T; 5, *Hfx. larsenii* JCM 13917^T; 6, *Hfx. lucentense* DSM14919^T; 7, *Hfx. prahovense* DSM 18310^T; 8, *Hfx. sulfurifontis* DSM 16227^T; 9. *Hfx. elongans* JCM 14791^T.

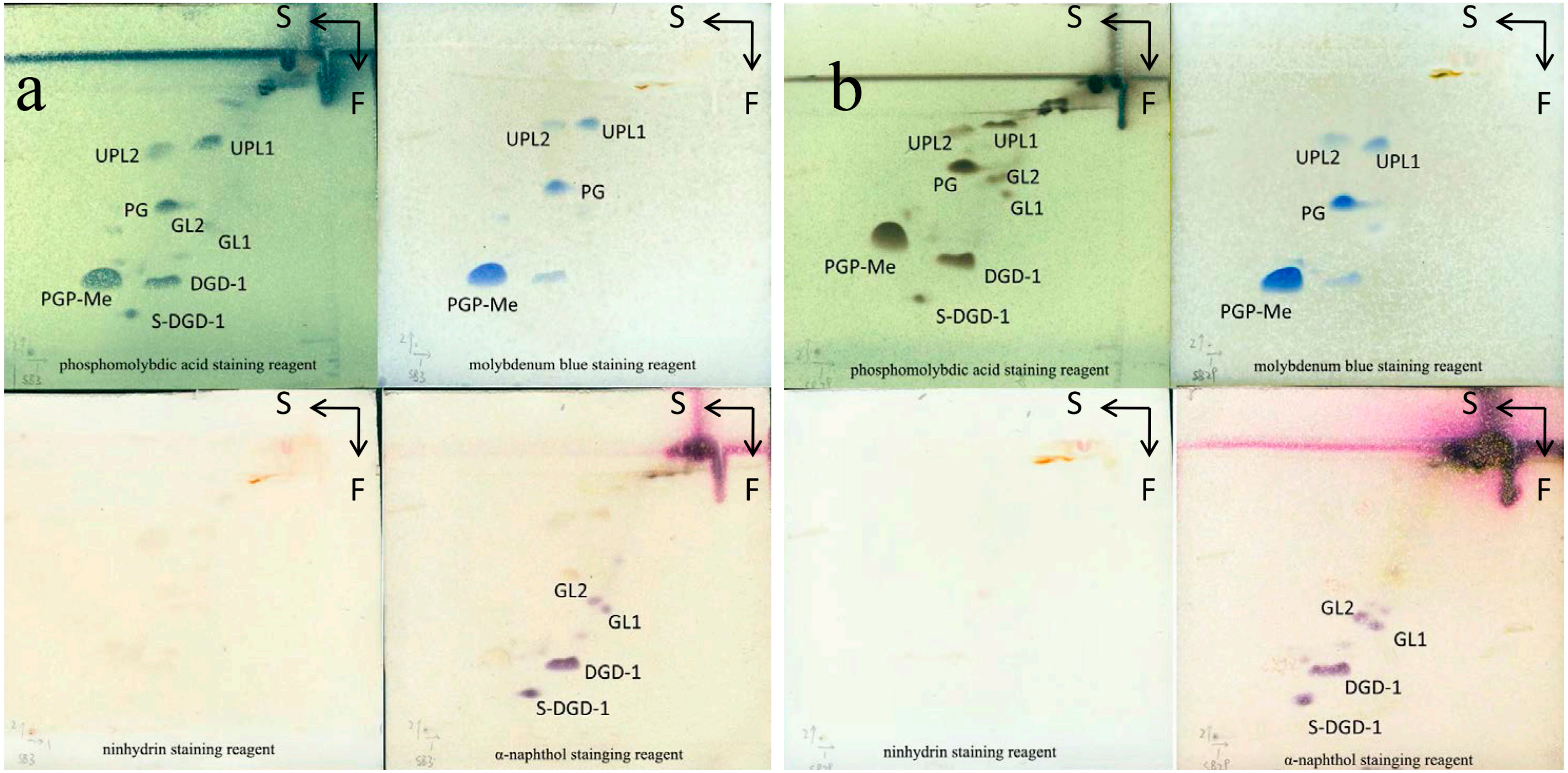

Figure S4. Polar lipids profiles for strain SB3^{T} (a) and SB29^{T} (b) include PG, PGP-Me, S-DGD-1, DGD-1, UPL1 and UPL2; and an unidentified GL, which support the placement of both strains into the genus *Haloferax*. The TLC plates were stained with 10% phosphomolybdic acid in 95% ethanol for detection of total lipids, 0.2 % ninhydrin in 1-butanol for aminolipids, molybdenum blue reagent (Sigma) for phospholipids, and a-naphthol staining reagent (Sigma) for cholines. Abbreviations: PG, phosphatidyl glycerol; PGP-Me, phosphatidyl glycerol phosphate-methlester; S-DGD-1, sulfated mannosyl glucosyl diether; DGD-1, mannosyl glucosyl diether; UPL1 and UPL2, two unidentified phospholipids; GL, glycolipid; F, first dimension of TLC; S, second dimension of TLC.

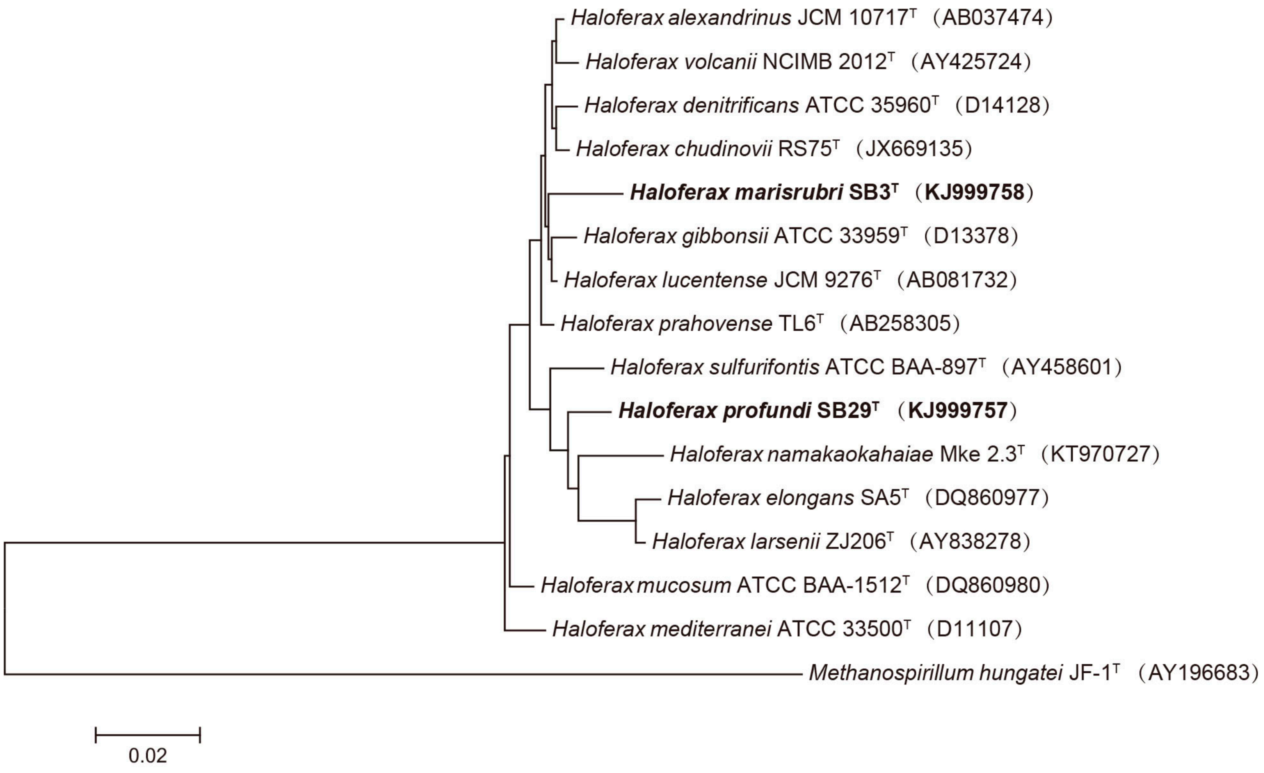

Figure S5. Neighbour-joining phylogenetic tree based on 16S rRNA gene sequences showing the relationships between strain SB3^{T} and SB29^{T} (bold) and related taxa. Scale bar indicates 0.01 substitutions per nucleotide position. *Methanospirillum hungatei* JF-1^{T} (AY196683) was used as outgroup. GenBank accession numbers are indicated for each strain in parentheses.

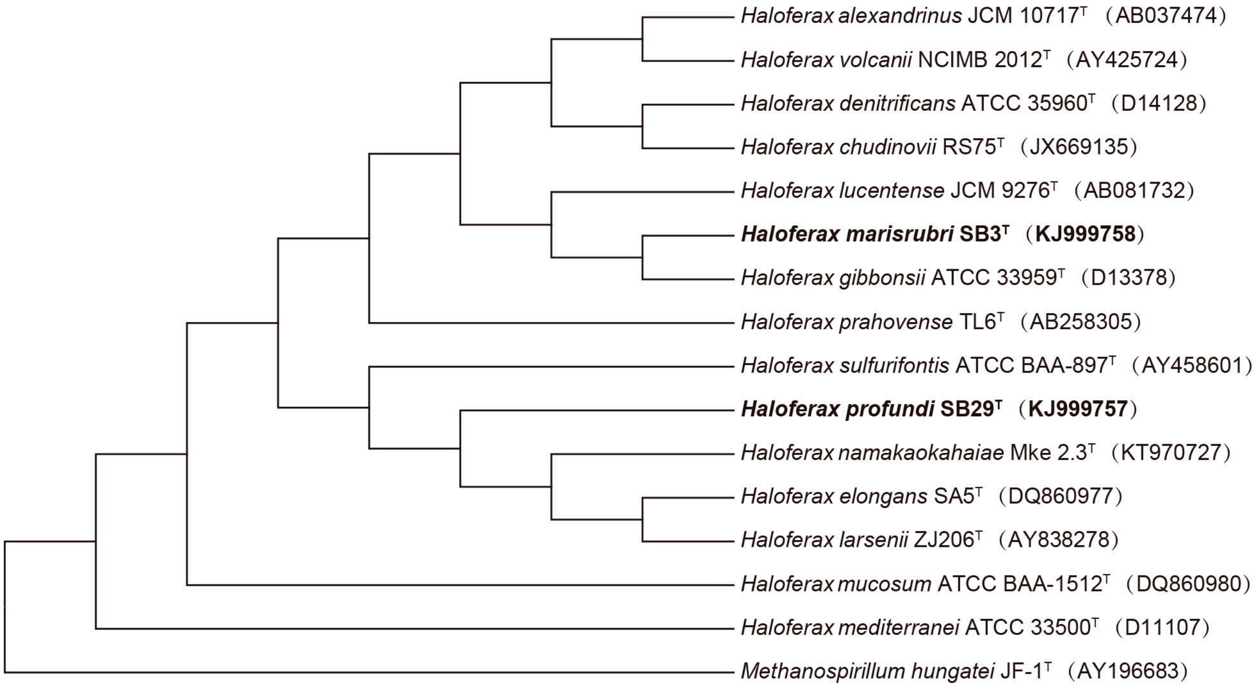

Figure S6. Maximum parsimony phylogenetic tree based on 16S rRNA gene sequences showing the relationships between strain SB3^{T} and SB29^{T} (both in bold) and related taxa. *Methanospirillum hungatei* JF-1^{T} (AY196683) was used as outgroup. GenBank accession numbers are indicated for each strain in parentheses.

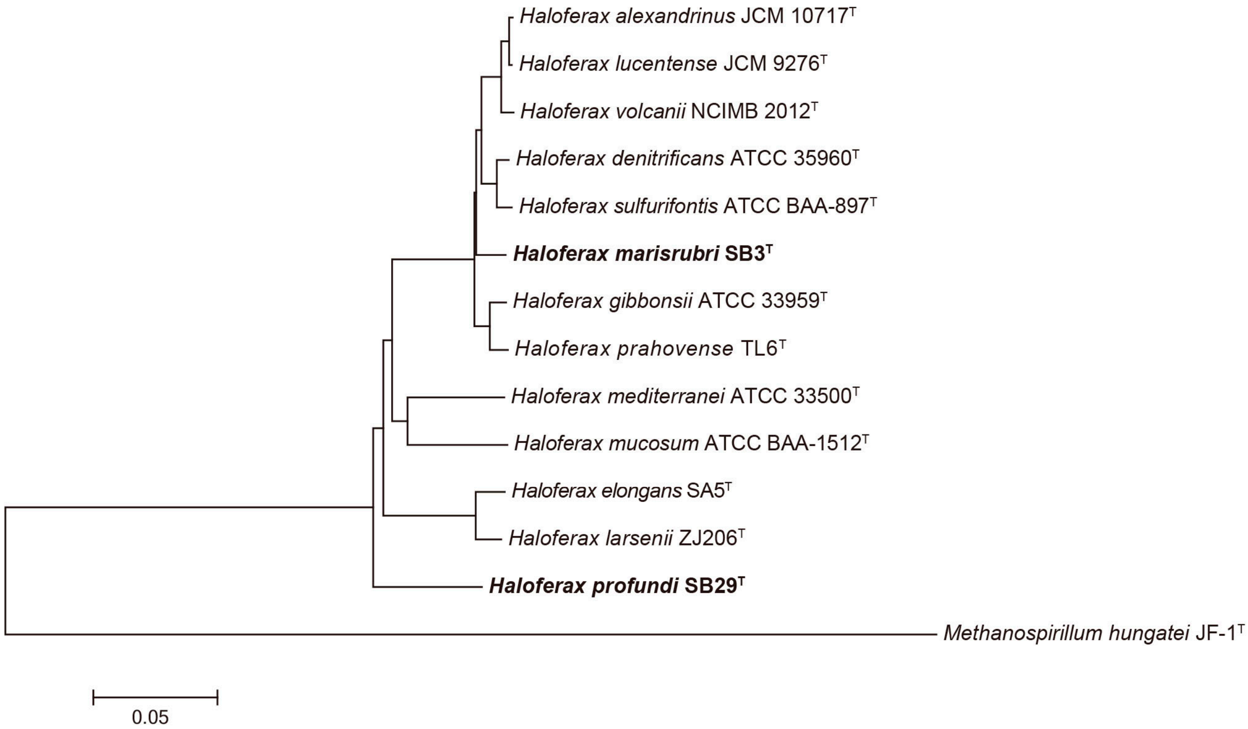

Figure S7. Phylogenomic tree based on the concatenation of the 31 single-copy core genes from all 11 available genomes of type strains for *Haloferax* spp. were inferred with Neighbor-Joining (NJ) method and rooted by *Methanospirillum hungatei* JF-1^{T}. The genes were katG2, top6A, ntpB, atpA, ftsZ_2, aroB, fusA, uvrA, Ion, gatA, ndk, rplB, ftsZ_1, pdxS, nikR_2, guaA_2, rpoB_1, rpsK, rpsJ_1, tufB_1, rps12P, rps28e, DUF2073, rps2P, rpoK, rpl40e, rps3p, Mcm2 and three hypothetical proteins (PRK00939, PRK04016, PRK04243; https://www.ncbi.nlm.nih.gov/cdd) as determined in Roary (see methods section).

Table S1. Average nucleotide identity (ANIm) and digital DNA-DNA hybridization (dDDH) values (%) between the genomes of strain SB3^T (GenBank accession: LOPW00000000) , and closely related genomes in GenBank.

Most closely related genomes in GenBank	GenBank assembly accession	ANIm	DDH
Haloferax gibbonsii ATCC 33959^T	GCA_000336775.1	92.2	49.2
Haloferax mediterranei ATCC 33500^T	GCA_000306765.2	81.8	26.0
Haloferax denitrificans ATCC 35960^T	GCA_000337795.1	92.6	51.1
Haloferax sulfurifontis ATCC BAA-897^T	GCA_000337835.1	91.9	48.7
Haloferax mucosum ATCC BAA-1512^T	GCA_000337815.1	81.8	25.9
Haloferax lucentense JCM 9276^T	GCA_000336795.1	92.0	49.2
Haloferax alexandrinus JCM 10717^T	GCA_000336735.1	92.0	49.4
Haloferax volcanii NCIMB 2012^T	GCA_000025685.1	91.9	48.8
Haloferax elongans SA5^T	GCA_000336755.1	80.3	24.3
Haloferax profundi SB29^T	GCA_001469865.1	81.1	25.1
Haloferax prahovense TL6^T	GCA_000336815.1	92.2	50.0
Haloferax larsenii ZJ206 ^{T}	GCA_000336955.1	80.5	24.5

Table S2. Average nucleotide identity (ANIm) and digital DNA-DNA hybridization (dDDH) values (%) between the genomes of strain SB29^T (GenBank accession no.: LOPV00000000), and closely related genomes in GenBank. .

Most closely related genomes in GenBank	GenBank assembly accession	ANIm	DDH
Haloferax gibbonsii ATCC 33959^T	GCA_000336775.1	80.8	25.0
Haloferax mediterranei ATCC 33500^T	GCA_000306765.2	80.7	25.1
Haloferax denitrificans ATCC 35960^T	GCA_000337795.1	80.8	25.1
Haloferax sulfurifontis ATCC BAA-897^T	GCA_000337835.1	82.6	28.1
Haloferax mucosum ATCC BAA-1512^T	GCA_000337815.1	80.2	25.6
Haloferax lucentense JCM 9276^T	GCA_000336795.1	80.7	25.0
Haloferax alexandrinus JCM 10717^T	GCA_000336735.1	80.7	24.8
Haloferax volcanii NCIMB 2012^T	GCA_000025685.1	80.7	24.9
Haloferax elongans SA5^T	GCA_000336755.1	80.1	24.3
Haloferax marisrubri SB3^T	GCA_001469875.2	81.0	25.1
Haloferax prahovense TL6^T	GCA_000336815.1	80.8	25.0
Haloferax larsenii ZJ206 ^{T}	GCA_000336955.1	81.4	25.5
